# Hysterosalpingography findings in infertile Sudanese women: a cross-sectional study on tube blockage

**DOI:** 10.11604/pamj.2024.48.62.39517

**Published:** 2024-06-18

**Authors:** Eiman Kamal, Maisa Elzaki

**Affiliations:** 1 Faculty of Radiological Sciences and Medical Imaging, Alzaiem Alazhari University, Khartoum, Sudan

**Keywords:** Hysterosalpingography, secondary infertility, primary infertility, pelvis inflammatory disease

## Abstract

**Introduction:**

infertility is a significant public health concern in Africa and Hysterosalpingography (HSG) is an affordable option for initial treatment. This study aimed to provide information about the incidence of abnormal pathology and tubal findings in HSG of Sudanese women who experienced infertility.

**Methods:**

this prospective cross-sectional study included 100 infertile patients who were requested for HSG, including age, duration of infertility, body mass index (BMI), medical history, and HSG findings collected after performing the radiographic test, which was diagnosed by an experienced radiologist.

**Results:**

one hundred infertile women (46% and 54%) experienced primary and secondary infertility, respectively. Mean age was (31.1 ± 5.2, 27.5 ± 6.0) years, and BMI was (25.1 ± 3.3, 25.7 ± 2.9) Kg/cm^2^ for primary and secondary infertility respectively. Abnormal findings prevalence was (29/46, 63%) and (30/54, 56%). The incidence of fallopian tube abnormality was (52/100, 52% (25/46, 54.3%), and (27/56, 50%) for primary and secondary infertility, respectively. Forty-one percent of participants had normal hysterosalpingograms. Pelvic surgery was the highest risk factor in 24% of the participants. Age and medical history were significantly associated with the infertility type (P < 0.05).

**Conclusion:**

infertile patients who underwent hysterosalpingography (HSG) were predominantly older, with secondary infertility being slightly more common, underscoring the importance of early diagnostic evaluation and care. Fallopian tube abnormalities were the most common cause of infertility, with tube blockage affecting nearly half of the participants. Additionally, this study revealed that prior pelvic surgery significantly increased the risk of infertility.

## Introduction

Infertility, defined as the inability of women to perform clinical childbearing after one year of sexual communication [1,2], poses a significant challenge to women worldwide. Hysterosalpingography (HSG) is a pivotal radiological tool for diagnosing and evaluating infertility, particularly when assessing the condition of the fallopian tubes [3]. Widely regarded as the gold standard for fallopian tube assessment, HSG provides critical insights into tubal patency and morphology [4,5]. As a result, the correct diagnosis of the fallopian tubes, whether they are blocked, which failed to pass contrast media and spill into the peritoneal cavity [4], or are enlarged in the ampullary section without spillage [1,6]. Physicians also recommend it to examine the uterine cavity´s abnormalities [6]. The application of HSG to identify tubal blockage is accurate, with 94% sensitivity and 92% specificity [1].

Approximately 10-15% of couples worldwide suffer from infertility, which is the most common health problem among women in developing countries [1,7] where the lack of education and general health of women of childbearing age further exacerbates this issue in Africa [1]. A study from Nigeria underscores that the prevalence of primary infertility is higher with a high incidence of tubal blockage due to infection and inflammation [6]. According to a study conducted by Al Subhi *et al*. tubal blockage causes infertility [8]. Another study revealed that the most common cause of tubal blockage is pelvic inflammatory disease (PID), which can result from previous abdominal or pelvic surgeries [8] or sexually transmitted infections such as chlamydia [8,9]. Consequently, the etiology of infertility varies across geographic regions and is influenced by local socioeconomic and healthcare factors.

The high prevalence of infertility among women in developing countries, particularly in Africa, is exacerbated by a lack of education and general health issues, with many notable causes. Consequently, there is a critical need to understand the specific is critical for understanding the specific causes of infertility in the Sudanese population.

By interpreting hysterosalpingography (HSG) findings related to infertility, this study aimed to provide valuable insights into the prevalence of pathological and tubal findings of HSG among Sudanese women experiencing infertility. By shedding light on these aspects, our study sought to enhance the understanding of infertility in the Sudanese population and cover ways for more informed interventions to address this pressing healthcare concern.

## Methods

**Study design and setting:** this prospective cross-sectional study was conducted at Friendship Hospital Omdurman in Khartoum, Sudan, from February to July 2021.

**Study population:** this study included 100 Sudanese women with a history of primary or secondary infertility scheduled to undergo HSG. The sample size was randomly selected and not calculated, and no patient withdrew from the study. Patients who had undergone previous infertility treatments or sterilization procedures were excluded from the study.

**Data collection:** prior to the HSG procedure, the participants were interviewed to gather demographic details, such as age and relevant clinical information, including the duration of infertility and medical history. Interviews were conducted in a private and comfortable setting to facilitate open communication and information exchange.

**Sample collection and Radiographic examination:** HSG examinations were conducted under the supervision of experienced radiologists by a radiologist, radiographer, and nursing staff. Patients were fully informed about the procedure and potential complications beforehand and provided informed consent once they arrived at the X-ray department and were scheduled during the proliferative phase of the menstrual period [10]. For patients with irregular cycles, a pregnancy test was performed prior to the procedure. During the examination, patients were placed in a lithotomy position, aseptic techniques were used to maintain a sterile environment, and a vaginal speculum was inserted, followed by slow injection of contrast media under fluoroscopic imaging, with films taken to visualize the uterine cavity and any spillage into the peritoneal cavity.

**Definitions of variables:** the variables in the study included the patient´s demographic data, including age and body mass index (BMI) calculated after taking the patient´s height and weight as the formula (BMI = weight (kg) / height^2^ (m^2^)) [11], and clinical data, including duration of infertility, and medical history all this data was collected through interviewing the patient and registered on the datasheet and the another collected variables were HSG findings taken from the radiographic report written by the experienced radiologist to conclude the pathological findings, this variable classified and itemized on data collecting sheet. To address potential sources of bias, we standardized data collection protocols, ensured interviewers were thoroughly trained, utilized blinded and multiple reviews for HSG findings, and implemented regular quality checks and statistical adjustments.

**Statistical analysis:** the data were analyzed using the IBM Statistical Package for the Social Sciences (SPSS) version 25.0. Descriptive statistics, frequencies, and proportions were used to characterize categorical variables, whereas continuous variables were defined using the mean and standard deviation. Nonparametric tests: the chi-squared test (X^2^) was used to determine the association between the type of infertility and independent variables, and continuous variables were compared using the student´s t-test, and no missing data needed to be addressed. Differences were considered statistically significant at P < 0.05, with a 95% confidence interval.

**Ethical considerations:** ethical approval was obtained from the research committee of Al-Zaiem Al-Azhari University, Faculty of Radiological Science and Medical Imaging in Khartoum, Sudan. Written informed consent was obtained from each participant, and all patient data were coded to preserve confidentiality.

## Results

**Demographic data:** this study containing 100 women whose mean age was significantly higher (p-value < 0.05) in the secondary infertility group (31.1 years ± 5.2) compared to the primary infertility group (27.5 years ± 6.0). The distribution of age groups also differed, with a higher proportion of women in the secondary infertility group falling into the 33-39-year-old range (44.4%) than in the primary infertility group (17.4%). The average duration of infertility was similar between both groups (4.7 years ± 3.5 in primary and 5.0 years ± 3.2 in secondary). However, the distributions across different time frames showed some variations.

The primary infertility group had a higher percentage of women experiencing infertility for 0-2 years (34.8%) than did the secondary infertility group (20.4%). Conversely, the secondary infertility group had a higher proportion of women experiencing infertility for 3-5 years (44.4%) than did the primary infertility group (32.6%). No statistically significant difference was observed in overall BMI between the two groups (25.1 ± 3.3 Kg/cm^2^ in primary and 25.7 ± 2.9 Kg/cm^2^ in secondary). However, the distribution of the BMI categories differed slightly. The primary infertility group had a higher percentage of women with a normal BMI (18.5 - 24.9 Kg/cm^2^) (54.3%) than the secondary infertility group (40.7%). Conversely, the secondary infertility group had a slightly higher prevalence of overweight women (25-29.9 Kg/cm^2^) women (46.3%) than the primary group (30.4%) (Table 1).

**Table 1:** demographic data and duration of infertility cross-tabulated by infertility type

Variables	Primary infertility		Secondary infertility		Total	
	N=46	100%	N=54	100%	N=100	100%
**Age (year)**	**27.5 ± 6.0 (20-45)**		**31.1 ± 5.2 (21-40)**		**29.6 ± 5.8 (20-45)**	
20-27	25	54.3%	13	24.1%	38	38%
28-32	11	23.9%	16	29.6%	27	27%
33-39	8	17.4%	24	44.4%	32	32%
>40	2	4.3%	1	1.9%	3	3%
**Duration of infertility (years)**	**4.7 ± 3.5 (0.5-18)**		**5.0 ± 3.2 (0.3-14)**		**4.9 ± 3.4 (0.3-18)**	
1-3 years	16	34.8%	11	20.4%	27	27%
4-5 years	15	32.6%	24	44.4%	39	39%
6-10 years	14	30.4%	15	27.8%	29	29%
>10 years	1	2.2%	4	7.4%	5	5%
**BMI (kg/m^2^)**	**25.1 ± 3.3 (19-31.9)**		**25.7 ± 2.9 (18.8-32.5)**		**25.5 ± 3.1 (18.8-32.5)**	
Normal (18.5-24.9)	25	54.3%	22	40.7	47	47%
Overweight (25-29.9)	14	30.4%	25	46.3%	39	39%
Obesity>30	7	15.2%	7	13.0%	14	14%

P-value < 0.05 considered significant different; BMI: body mass index

**Hysterosalpingography findings:** to evaluate the prevalence of abnormal findings on hysterosalpingography (HSG) examinations (Table 2), 46% of women were diagnosed with primary infertility and 54% with secondary infertility. Abnormal findings were identified on HSG in 59 women (59%). No significant difference was observed in the prevalence of abnormal HSG findings between the primary (29/46, 63%) and secondary infertility (30/54, 56%) groups (p > 0.05).

**Table 2:** hysterosalpingography (HSG) and tubal findings and medical history according to infertility type

Type of Infertility	Primary infertility		Secondary infertility		Total	
Total	N=46	100%	N=54	100%	100	
**HSG findings**						
Bilateral tubal blockage	9	19.6%	8	14.8%	17	17.0%
Rt tube blockage	9	19.6%	9	16.7%	18	18.0%
Lt tube blockage	6	13%	7	13.0%	13	13.0%
Hydrosalpinges	1	2.2%	3	5.6%	4	4.0%
No findings	17	36.9%	24	44.4%	41	41.0%
Uterine abnormality	4	8.6%	3	5.5%	7	7.0%
**Medical history and risk factors**						
No medical history	22	47.8	20	37.0%	42	42%
Thyroid Abnormality	5	10.9%	12	22.2%	17	17%
PID	10	21.7%	6	11.1%	16	16%
Pelvic surgery	9	19.6%	15	27.8%	24	24%
TB	0	0.0%	1	1.9%	1	1.0%

P-value < 0.05 is considered a significant difference; PID: pelvic inflammatory disease; TB: tuberculosis; Rt: right; Lt: left

Fallopian tube abnormalities were the most common type of abnormality, identified in 53 (53%) women. This category included bilateral tubal blockage, which was observed in 17 women (17%) (9/46, 20%) in the primary (8/54, 15%) and in the secondary infertility group (p > 0.05). Unilateral tubal blockage was identified in 31 women (31%), with equal distribution between right (18/46 in primary and 9/54 in secondary) and left (13/46 in primary and 7/54 in secondary) tubal blockages. Only four women (4%) had hydrosalpinges, with a slightly higher prevalence in the secondary infertility group (3/54) than in the primary infertility group (1/46); however, the difference was not statistically significant (p > 0.05). Uterine abnormalities, such as polyps, were identified in seven women (7%), with no significant differences in prevalence between the primary (4/46) and secondary infertility (3/54) groups (p > 0.05). Normal HSG findings, indicating no apparent abnormalities, were observed in 41 (41%) women. This was higher in the secondary infertility group (24/54, 44%) than in the primary infertility group (17/46, 37%); however, the difference was not statistically significant (P > 0.05) (Table 2).

**Medical history and risk factor:** as shown in Table 2, women with a medical history of thyroid abnormalities were (12, 22.2%) infertility and (15, 27.8%), and prior pelvic surgery (5, 10.9%) and (9, 19.6%), in secondary and primary infertility respectively. Only one case of tuberculosis (TB) was identified in the secondary infertility group. Pelvic inflammatory disease (PID) and no history of relevant medical conditions were more common in the primary infertility group (10, 21.7%) and (22, 47.8%), respectively, than in the secondary infertility group (6, 11.1%) and (20, 37.0%) respectively. Forty-two percent of patients had no previous medical history.

**Association of variables and infertility findings:** the results of the analysis revealed no statistically significant differences (P > 0.05) between BMI and infertility type (Table 3). In this study, there was a significant association (P = 0.002) between infertility type and age and a significant correlation (P = 0.004) between infertility type and medical history and risk factors (Table 3).

**Table 3:** relationship between age group, medical history, and body mass index (BMI) with infertility type

	Age group	Risk factors and medical history	BMI
Pearson Chi-Square	0.276	0.752	2.671
P- value	0.002*	0.004*	0.263

* P- value < 0.05 is considered a significant different; BMI: body mass index

## Discussion

A hysterosalpingogram is a noninvasive test that can be used to detect abnormalities in the cervix and uterus but is more focused on evaluating fallopian tube patency. This study aimed to offer useful information about the occurrence of pathological and tubal abnormalities in (HSG) among Sudanese women who are facing infertility issues.

Our study revealed that secondary infertility is more common than primary infertility and the prevalence of infertility is higher among women aged 20 - 27 years. Although primary infertility affects women younger than 27 years of age, secondary infertility is more prevalent in women aged 33 years or older. Tubal abnormalities, particularly blockage and hydrosalpinx, have been identified as major causes of infertility, particularly in patients with secondary infertility. Additionally, medical history, such as a medical history of thyroid abnormalities, prior pelvic surgery, and pelvic inflammatory disease, significantly contributed to infertility.

The global prevalence of infertility varies, with secondary infertility being more common than primary infertility in several regions. Studies [1,2,5,9,12,13] have shown that this trend is consistent with earlier research, likely because secondary infertility occurs during the peak reproductive years of a woman. In accordance with these data, most cases within this cohort, in contrast to the conclusions of Adedigba *et al*. who reported that the age group of 31 - 35 years was the most primary infertile category [5].

However, in this study, the most prevalent among those over the age of 33 years was secondary infertility, with a mean duration of infertility of 5.0 ± 3.2 years comparable to 1 - 4 years in Ghanian women [2], which may be due to women in Sudan married in the mid-twenties, leading to longer time duration. Moreover, many women in their thirties, many of them had prior pregnancies and had difficulty conceiving again due to changes in reproductive health.

Primary infertility affects patients under the age of 27 years and more than 5 years of infertility duration, a similar trend was drawn by Abdullah *et al*. [13,14], who stated that the advanced age of the woman and the long duration of marriage reduces their ability to reproduce and to have a new child.

Concerning participants´ body mass index, almost half of the patients (48%) had a normal BMI (18.5-24.9 kg/m^2^); therefore, the remaining patients (52%) were overweight (25-29.9 kg/m^2^) or obese. Obesity poses significant challenges for conception, primarily owing to its impact on ovulation and menstrual regularity [15]. Investigating the intricate pathways of reproductive health, our research examined the crucial role that fallopian tube obstruction plays in infertility. Our study aligns with established studies [9,12,16], and offers additional insights into this issue. Specifically, our radiographic observations highlight tube obstruction identified through HSG examination.

In the sample analyzed, both unilateral and bilateral tube blockage was deemed the most definitive finding, which corroborates previous research [2,6,9]. The researchers concluded that “tubal abnormalities (obstruction, dilation) were the most prevalent reasons for infertility” [9]. In this study, total bilateral obstruction was observed in 17% of all cases and accounted for 14.8% of cases of secondary infertility. Therefore, this was less common than tubal blockage in primary infertility. Whereas Bello *et al*. discovered a greater prevalence of tubal abnormalities in patients with secondary infertility [1]. The total number of tubal abnormality cases was frequent in women with secondary infertility, which might be explained by the potential for inadequate health care after birth or abortion as well as an increased risk of PID [1].

An interesting aspect that emerged from the analysis was that the prevalence of the right blockage tube was higher than that of the left side (18% vs 13%). Similar results were reported in an investigation by Adedigba *et al*. who explained that due to its possibility that because the right fallopian tube is near the appendix and cecum [5]. Onwuchekwa *et al*. confirmed that there was increased obstruction in the right-side tube due to previous pelvic inflammation and adhesions [6].

**Figure 1:**
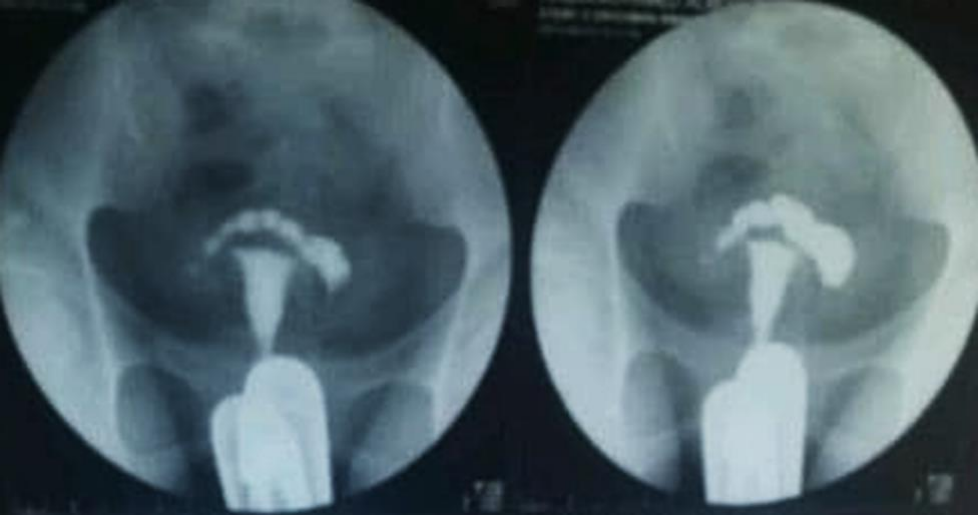
hydrosalpinx with dilation of the blocked fallopian tube

Remarkably, the presence of a hydrosalpinx (Figure 1) emerged as another key finding in the HSG scan used to examine the fallopian tubes. In previous study observed that hydrosalpinx was identified as the high leading cause of infertility resulting from tubal pathology [1], which contrasts with the prevalence findings of this study, which showed the lowest incidence of tubal pathology as a cause of infertility and was more prevalent in secondary infertility than in primary infertility. This is consistent with a previous study [5], which indicated that secondary infertility is twice as prevalent among patients with all types of hydrosalpinx as compared to primary infertility.

Upon comparing the prevalence of hydrosalpinges as a discovery of infertility in the Sudanese population in this study, it was found that they constituted only 4% of all cases. This figure was lower than the prevalence of hydrosalpinges in women from sub-Saharan Africa [7], which is reported (12.6%) and (12.4%) of the Nigerian population [5], and reported a rate of 14.5% among Ghanaian women [2,12]. The presence of the hydrosalpinges is associated with an increased risk of inflammation, which can ultimately result in tubal blockage.

In this research, women with suspected blocked fallopian tubes, 24% of the total individuals had a history of abdominal or pelvic surgeries, along with other risk factors. Based on a study by Toufig *et al*. [14] report of all participants diagnosed with blocked tubes, underscoring the high prevalence of this condition as a leading cause of infertility in Sudan. These results are consistent with previous studies [14,17] that have explored the etiology of fallopian tube blockage in other populations due to sepsis, postoperative inflammation [14], and adhesions [15] that affect the peritoneal cavity and may affect the fallopian tubes. PID also plays a role in infertility [16], more clearly in patients with secondary infertility, as observed in this study, whereas previous studies confirmed that PID was a common cause of infertility [16] in the Sudanese nation [13,14]. The risk of endocrine and thyroid diseases has been reported to primarily occur in patients with secondary infertility, as reported by Toufig *et al*. These patients' medical history significantly correlates with the type of infertility they experience [14].

**Figure 2:**
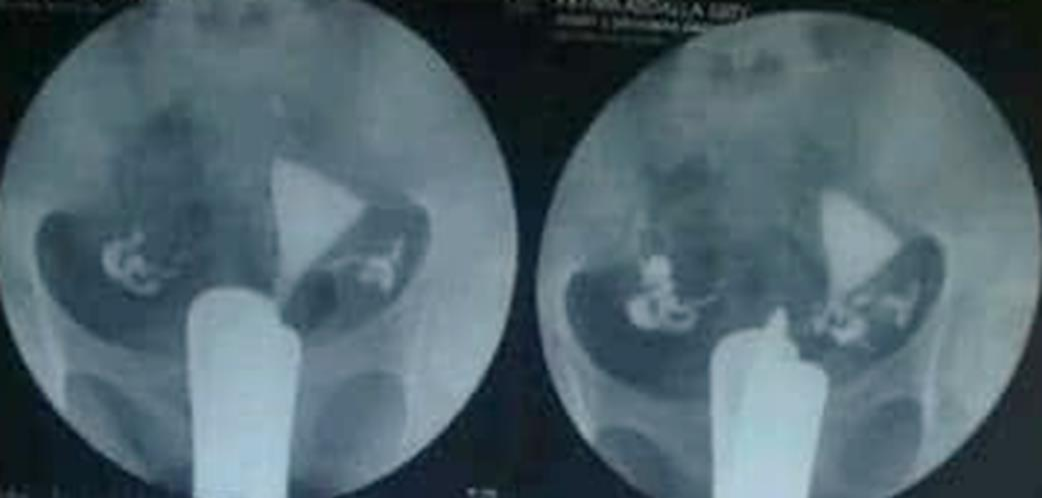
normal hysterosalpingography (HSG) exam with normal uterus and contrast spill

The indistinguishable outcomes showed that approximately 41% of infertile women showed normal findings on HSG examination, a result that aligns with another study conducted in Sudan [14], which found that more than half of the infertile women exhibited normal HSG results (Figure 2). This finding was further validated by a study conducted in Nigeria, which arrived at a similar conclusion [6], our findings align with those of our findings support Aziz *et al*. who stated that “the majority of patients with primary infertility had normal HSG findings, indicating that the etiology was not physical” [9].

The discovery of only one case of tuberculosis (TB) in the secondary infertility group compared to none in the primary infertility group suggests a potential link between secondary infertility and the reactivation of latent TB, a condition in which bacteria remain dormant in the body after a previous infection. Pregnancy and the postpartum period can suppress the immune system, potentially increasing the risk of TB reactivation [18]. This could explain why TB was observed only in the secondary infertility group, which by definition had previously been conceived.

Conducting thorough medical evaluations and addressing underlying health issues are crucial for optimizing fertility outcomes. In Sudan, infertility is recognized as a significant public health concern, necessitating the implementation of comprehensive strategies for its prevention, diagnosis, and treatment. It is essential to address both primary and secondary infertility, as well as associated risk factors, such as obesity and pelvic infections, through a multifaceted approach that involves healthcare providers, policymakers, and public health interventions.

These findings may not be representative, and the reality of the results may not be explained by the small sample size and cannot be generalized because it was selected from a single hospital center which may not reflect the diversity of the broader population.

## Conclusion

This study sheds light on the hysterosalpingography findings on the complex issue of infertility in Sudanese women, particularly noting the high rates of secondary infertility and the significant roles played by age, obesity, and tubal blockage. This finding emphasizes the critical need for early detection and intervention. This research advocates a holistic approach to reproductive health, which includes early screening, precise diagnosis, and individualized treatments. Furthermore, it calls for preventive measures, educational initiatives, and better access to quality reproductive health services to reduce the prevalence of infertility associated with tubal problems.

### What is known about this topic

- *It is widely recognized that secondary infertility is more prevalent than primary infertility;*
- *Pelvic inflammatory disease (PID) is a potential causative factor for both hydrosalpinges and tubal blockages*.

### What this study adds

- *This study contributes to the understanding of the primary cause of infertility in Sudan and the primary risk factors that hinder women from becoming pregnant;*
- *This study presents vital information regarding the frequency of tubal obstruction as a contributing factor to infertility;*
- *The following text underscores the potential implications of insufficient healthcare in the reproductive system, specifically the heightened likelihood of developing pelvic inflammatory disease (PID) and consequential tubal abnormalities*.
